# African animal trypanocide resistance: A systematic review and meta-analysis

**DOI:** 10.3389/fvets.2022.950248

**Published:** 2023-01-04

**Authors:** Keneth Iceland Kasozi, Ewan Thomas MacLeod, Susan Christina Welburn

**Affiliations:** ^1^Infection Medicine, Deanery of Biomedical Sciences, College of Medicine and Veterinary Medicine, University of Edinburgh, Edinburgh, United Kingdom; ^2^School of Medicine, Kabale University, Kabale, Uganda; ^3^Zhejiang University-University of Edinburgh Institute, Zhejiang University School of Medicine, Zhejiang University, Haining, China

**Keywords:** African animal trypanosomiasis, *Trypanosoma brucei* brucei, trypanocide resistance, drug resistance, *T. evansi*, *T. congolense*, *T. vivax*, bovine trypanosomiasis

## Abstract

**Background:**

African animal trypanocide resistance (AATr) continues to undermine global efforts to eliminate the transmission of African trypanosomiasis in endemic communities. The continued lack of new trypanocides has precipitated drug misuse and overuse, thus contributing to the development of the AATr phenotype. In this study, we investigated the threat associated with AATr by using the major globally available chemotherapeutical agents.

**Methods:**

A total of seven electronic databases were screened for an article on trypanocide resistance in AATr by using keywords on preclinical and clinical trials with the number of animals with treatment relapse, days taken to relapse, and resistant gene markers using the PRISMA checklist. Data were cleaned using the SR deduplicator and covidence and analyzed using Cochrane RevMan®. Dichotomous outputs were presented using risk ratio (RR), while continuous data were presented using the standardized mean difference (SMD) at a 95% confidence interval.

**Results:**

A total of eight publications in which diminazene aceturate (DA), isometamidium chloride (ISM), and homidium chloride/bromide (HB) were identified as the major trypanocides were used. In all preclinical studies, the development of resistance was in the order of HB > ISM > DA. DA vs. ISM (SMD = 0.15, 95% CI: −0.54, 0.83; *I*^2^ = 46%, *P* = 0.05), DA vs. HB (SMD = 0.96, 95% CI: 0.47, 1.45; *I*^2^ = 0%, *P* = 0.86), and HB vs. ISM (SMD = −0.41, 95% CI: −0.96, 0.14; *I*^2^ = 5%, *P* = 0.38) showed multiple cross-resistance. Clinical studies also showed evidence of multi-drug resistance on DA and ISM (RR = 1.01, 95% CI: 0.71–1.43; *I*^2^ = 46%, *P* = 0.16). To address resistance, most preclinical studies increased the dosage and the treatment time, and this failed to improve the patient's prognosis. Major markers of resistance explored include *Tb*AT1, P1/P2 transporters, folate transporters, such as F-I, F-II, F-III, and polyamine biosynthesis inhibitors. In addition, immunosuppressed hosts favor the development of AATr.

**Conclusion:**

AATr is a threat that requires a shift in the current disease control strategies in most developing nations due to inter-species transmission. Multi-drug cross-resistance against the only accessible trypanocides is a major public health risk, justifying the need to revise the policy in developing countries to promote control of African trypanosomiasis.

## 1. Introduction

African animal trypanosomiasis (AAT) is caused by infection with parasitic protozoa of the genus *Trypanosoma* (1). Major *Trypanosoma* species belongs to *T. brucei* (*T. b.) brucei, T. evansi, T. congolense*, and *T. vivax* [see Ref. (2) on AAT]. The major challenge associated with the elimination of AAT is the development of drug resistance, namely, African animal trypanocide resistance (AATr) (3–5). Previously, resistance was associated with mutations at particular loci in the pathogen's genome. For example, mutations in the *Tb*AT1 and purine transporters (P1/P2) were associated with an increased resistance phenotype in *T. brucei* isolates (6). Unfortunately, scant attention has been given to the development of novel therapies to address AATr with most funding being pledged for the control of human African trypanosomiasis (HAT). The World Health Organization (WHO) has a target of 2030 to eliminate the transmission of HAT [in particular, gHAT (7)]; however, livestock will continue to be sources of reinfection in endemic communities since these will continue to act as reservoirs of infection (8–10). In developing countries, especially in Africa, the continuous lack of capacity to develop novel therapies against AAT has led to a high reliance on foreign aid and little effort to promote local institutional research to explore novel therapeutical options and build capacity to invest and develop infrastructure in trypanosome chemotherapeutics (11, 12).

Major trypanocides used in Africa include diminazene aceturate (DA), isometamidium chloride (ISM), and homidium bromide/chloride (HB) (13). The established curative properties for DA are 3.5 mg/kg, ISM at 0.5 mg/kg, and HB at 0.02 mg/kg, all administered intramuscularly in cattle (14). The intramuscular route is preferred since most farmers in African farming communities do not have the crushes to effectively restrain the animals before drug administration, leading to the common practice of injecting animals while in motion within a traditional African village enclosure made of a fence (15). Commonly used trypanocides for prophylactic purposes are ISM/HB due to their slow release, leading to their common usage with DA to widen their spectrum of action. This is important since ISM is ineffective against *T. evansi* (16). DA absorption is mainly through the P2/*Tb*AT1 transporters, while ISM has also been associated with mitochondrial F1F0-ATP synthase activity (17, 18). Other less commonly used trypanocides in AAT include aminoquinaldine (quinapyramine sulfate), which interrupts mitochondrial activity in the parasites while melarsomine dihydrochloride arsenic is hydrophilic and highly trypanocidal (17, 19). Loss of P2/*Tb*AT1 receptors in the pathogen is a cornerstone of AATr. Mutations in the expression of mitochondrial proteins, namely, F1F0-ATP synthase, result in reduced ISM absorption by *Trypanosoma* species. Furthermore, increased expression of efflux pumps (20) is responsible for increased resistance although studies on this continue to be scarce. The objective of the current study was to quantify evidence on the development of AATr with an emphasis on preclinical and clinical studies.

## 2. Materials and methods

In this study, emphasis was placed on experimental and qualitative studies conducted on African animal trypanocide resistance. Electronic databases, such as EMBASE, PubMed, Web of Science, CABI Abstracts, and CABI Global Health, were searched for publications while gray literature from an advanced Google Search using the WHO, African Union, Food and Agriculture Organization (FAO), Organization for Animal Health [OIE, currently renamed World Organization for Animal Health (WOAH)], Drugs for Neglected Disease International (DNDI), Kenya Medical Research Institute (KEMRI), Coordinating Center for Trypanosomes in Uganda (COCTU), United States Center for Disease Control (CDC), and United States AID (USAID) websites were added. The following search terms used included: “African trypanosomiasis/ or T. *brucei*/ or trypanosomiasis/ or tsetse fly-borne diseases/ OR bovine trypanosomiasis or *Trypanosoma* or *T. brucei*).mp. OR tsetse fly.mp. OR glossina.mp.” AND “*T. brucei brucei*.mp. OR exp *T. vivax*/OR exp *T. congolense*/ OR *T. evansi*.mp” AND “trypanocides/ or diminazene/ or homidium bromide/ or isometamidium chloride/ or melarsomine/ or quinapyramine/AND trypano^*^ resistance” (see Supplementary material 1).

A total of 54 publications were acquired from the search, that is, 11 publications from Ovid, 2 from NCBI, 37 from Web of Science, and 4 from Google Search by searching several databases up to 4 January 2022 (Table 1).

**Table 1:** Papers retrieved from the search of each database.

**Sn**.	**Database**	**Source**	**Accessed**	**Papers imported to SR accelerator**
1.	CABI abstracts	OVID	1946-week 4 2021	3
2.	MEDLINE	OVID	1946-week 4 2021	3
3.	EMBASE	OVID	1976-week 51 2021	3
4.	CABI global health	OVID	1973-week 50 2021	2
5.	PubMed	NCBI	Week 50 2021	2
	Web of science	Clarivate	Week 50 2021	37
	Google search	DNDI	Jan 4, 2022	2
		CDC	Jan 4, 2022	1
		FAO	Jan 4, 2022	1
Total	54			

**Figure 1:**
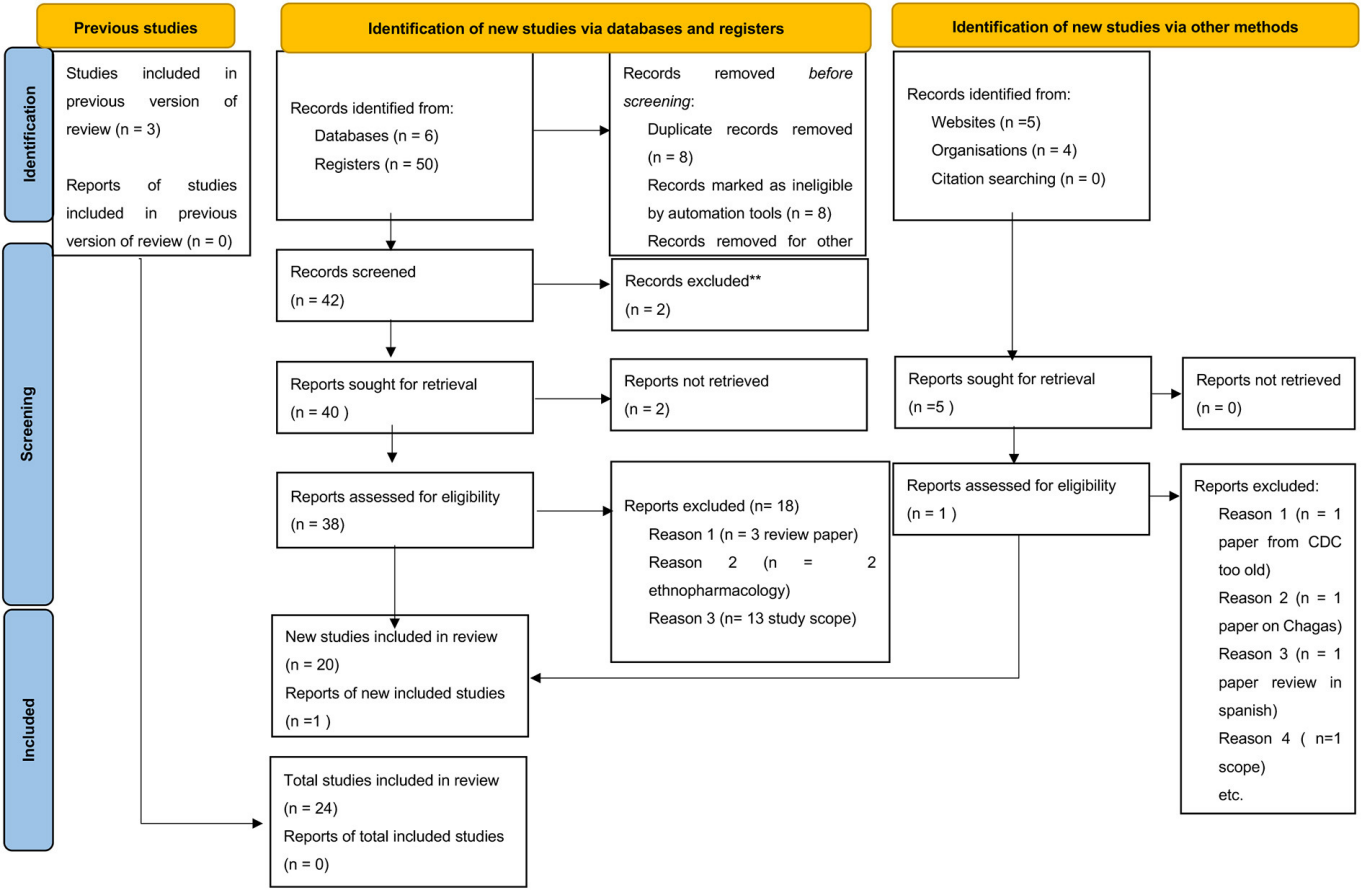
PRISMA checklist showing searched databases, registries, google search directories, and publications included in the study.

Publications acquired through this search (Table 1) were subsequently reviewed for relevance based on keywords, title, and abstract (by KIK and ETM). The selected publications were thoroughly screened by the SR deduplicator to remove duplications and scored for relevance by independent scholars (ETM and SCW). After resolving publication conflicts, the selected publications were searched for the full text and subsequent analysis using the PRISMA checklist (Figure 1). Furthermore, three more publications were identified by reviewing references in review articles and one from Google (*n* = 4), which were included among the papers eliminated during the data cleaning process (Figure 1) to generate a total of 54 research publications included in this systematic review. From the database registries, only 20 out of 50 publications were included after removing duplicates, ethnopharmacology articles, and review articles. From Google Search, only one out of four articles was added, while from the citation reviews within the review articles, three articles were included, leading to a total of 24 articles included for data extraction.

### 2.1. Statistical analysis

Data on the detection of AATr were presented as proportions, fixed, and random-effects models, and Begg's test and Egger's test were conducted to assess publication bias at a 95% confidence interval. Dichotomous data were analyzed using inverse variance at a 95% confidence interval, and the risk ratio was calculated using the fixed model, while continuous data were analyzed using standard mean difference, and random effect sizes were computed using RevMan® provided by Cochrane.

## 3. Results

### 3.1. Description of study articles by geographical distribution

A total of eight quantitative studies and 16 qualitative studies were identified in this study (Table 2). Only 4 out of 24 studies were cross-sectional studies that reported a prevalence of AATr ranging from 3.7% in Uganda (21) to 20.8% in Cameroon (41).

**Table 2:** Description of study publication on trypanosome pathogen, primary host, and location of study and prevalence.

**Study**	**Study type**	**Study design**	**Pathogen/gene target**	**Host**	**Location of the study**	**Sample size (*N*)**	**Prevalence (*n*, %)**
Afewerk et al. (22)	Quantitative	Cross-sectional study and experimental	*T. congolense*	Cattle *(B. indicus)*	Metekel Administrative Region, 550 km north west of Addis Ababa	484	83, 17.2%
Codjia et al. (23)	Quantitative	Experimental	*T. congolense*	East African Zebu *(B. indicus)* from Ghibe, Ethiopia	Kenya	NA	NA
Clausen et al. (24)	Quantitative	Experimental	*T. congolense*/*T. vivax*	Zebu *(B. indicus)*	Pastoral region of Samorogouan, Burkina Faso	NA	NA
Dagnache et al. (25)	Quantitative	Experimental	*T. vivax*	Zebu *(B. indicus)*	Jabitehenan district of Birsheleko area and Bahir Dar Zuria district in Ethiopia	NA	NA
Mulugeta et al. (26)	Quantitative	Experimental	*T. congolense*	Zebu *(B. indicus)* from Ghibe, Ethiopia	Kenya	NA	NA
Mungube et al. (5)	Quantitative	Cross-sectional	*T. congolense*	*B. indicus*	Sikasso Region, which is bordered to the east by Burkina Faso, Guinea Conakry to the west, to the south by Côte d'Ivoire and by Kouli- koro and Segou Regions to the north and north-east. The study was done in Mali.	796	125 (15.7%)
Tchamdja et al. (27)	Quantitative	Cross-sectional study	*T. congolense/T. vivax*	*B. indicus*	Kara and Savanes regions in Northern Togo	1,883	192, 10.2%
Mulandane et al. (28)	Quantitative	Cross-sectional study	*T. congolense*	*B. indicus*	Nicoadala district, Zambezia province of Mozambique	467	107
Mewamba et al. (29)	Qualitative	Experimental	*TbAT1*	Cattle sampled in this study belonged to breeds such as Zebu Goudali, Zebu White Fulani and Zebu Red Fulani with few crossbreeds (indigenous and exotic). The sheep were Djallonke west-African dwarfs, which are known to be trypanotolerant.	Yoko in the “Mbam et Nkim” Division of the center region of Cameroon	NA	NA
Koning & Jarvis (30)	Qualitative	Experimental	*P1/P2*	*T. b. brucei* from frozen stocks were grown in CD rats	United Kingdom	NA	NA
Carter et al. (31)	Qualitative	Experimental	*P2*	*Clones of T. b. brucei* S427	United Kingdom	NA	NA
Carruthers et al. (20)	Qualitative	Experimental	*T. b. congolense* folate transporters I, II, and III	Cultures of bloodstream forms of Savannah-type strain IL3000	United Kingdom	NA	NA
Bacchi et al. (32)	Qualitative	Experimental	Polyamine biosynthesis	*T. brucei rhodesiense* were obtained from A. R. Njogu of the Kenya Trypanosomiasis Research Institute (KETRI; Muguga, Kenya)	Switzerland	NA	NA
				Two *T. brucei rhodesiense* isolates were obtained from the American Type Culture Collection: ATCC 30119, the EATRO 105 strain isolated from a patient in Uganda in 1959, and ATCC 30027, the Wellcome CT strain isolated from a patient in 1934			
Zhang et al. (33)	Qualitative	Experimental	*T. evansi and T. equiperdum*	*T. evansi* were originally isolated in China, the Philippines, Ethiopia, Chad and Brasil	France	NA	NA
				The stocks of *T. equiperdum* originated from South Africa, China and the Pasteur Institute, Paris			
Gray & Peregrine (34)	Qualitative	Experimental	*T. congolense*	Clones from Tanzania, Burkina Faso, Ethiopia, and Kenya	United Kingdom	NA	NA
Anene et al. (35)	Qualitative	Experimental	*T. evansi stocks*	Cloned derivative of a camel isolate from Sudan	United Kingdom	NA	NA
Sahin et al. (36)	Qualitative	Experimetnal	*T. congolense* IL1180 *T. b. brucei*	*T. congolense* and *T. b. brucei* from Kenya	France	NA	NA
Zhang et al. (37)	Qualitative	Experimental	*T. evansi* and *T. equiperdum*	Bovine, canine, horse	France	NA	NA
Akpa et al. (38)	Qualitative	Experimental	*T. b. brucei*	These were obtained from Nigerian Institute for Trypanosomiasis Research (NITR), Vom, Plateau State, Nigeria in canines	Nigeria	NA	NA
Anene et al (39)	Qualitative	Experimental	*T.b. brucei*	Clinically infected dog that had relapsed following treatment with 2 doses of 7.0 mg/kg diminazene aceturate administered at 2-week intervals	Faculty of Veterinary Medicine, University of Nigeria	NA	NA
Osman et al. (40)	Qualitative	Experimental	*T. evansi*	Clones originally isolated from a camel naturally infected with Surra in the Sudan and known to be suramin resistant.	United Kingdom	NA	NA
Olila et al. (21)	Quantitative	Cross sectional and experimental	*T. brucei/T. vivax*	Dairy farms (*B. indicus*) in Mukono district, Uganda	Uganda	490	18 (3.7)
Mamoudou et al. (41)	Quantitative	Cross sectional and experimental	*T. congolense/T. brucei*	certain areas of the Adamaoua Department	Cameroon	221	46 (20.8)
Kulohoma et al. (4)	Qualitative	Experimental	TbAT1	Tsetse flies in Kwale county, Kenya	Kenya	546	20 (3.7)

Key: Quantitative studies reported prevalence while qualitative studies worked on trypanocide-resistant markers. Qualitative experimental studies directly involved laboratory animals by using trypanosome inoculum. NA, not applicable since this was an *in vitro* experiment.

**Figure 2:**
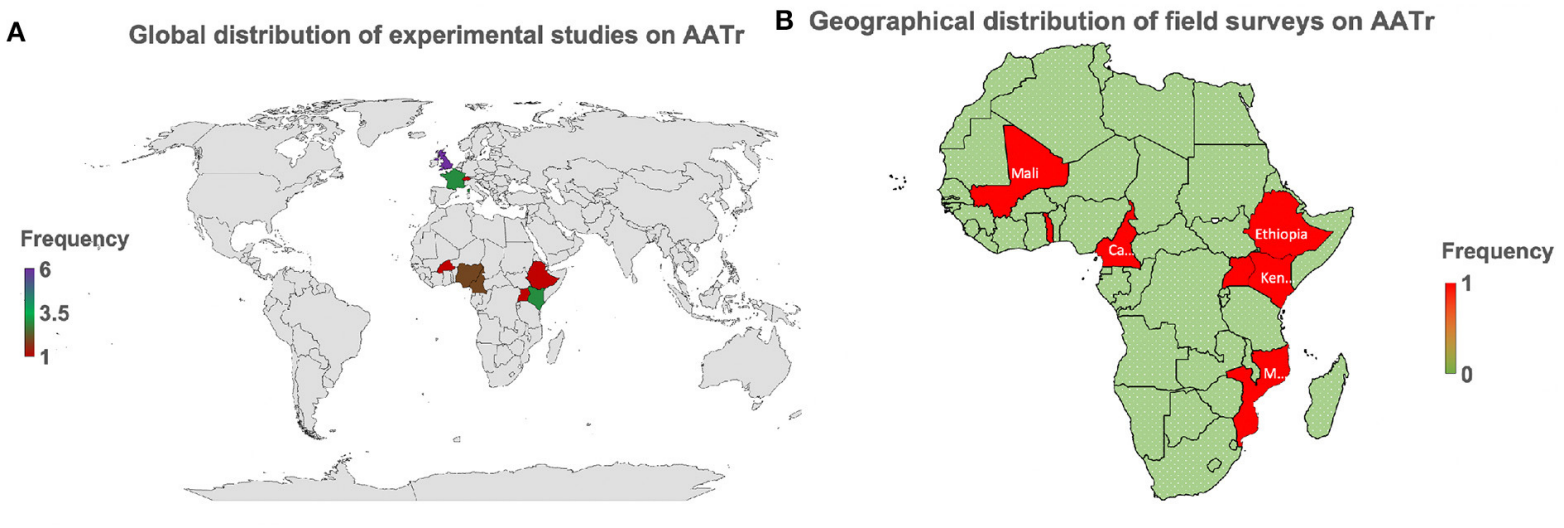
AATr, African animal trypanocide resistance. **(A)** This provides the global perspective showing that emphasis on AATr is in Africa and Europe. **(B)** Field surveys are only taking place in Africa globally demonstrating why AATr continues to be a neglected tropical disease. Biomedical experiments have generally been spearheaded by collaborative institutions in Europe while Africa continues to lag in this field due to the low number of active experimental studies on the African continent on AATr **(A)**. This has subsequently translated to a continued lack of commitment by local African institutions to routinely conduct surveillance activities to monitor the development of drug resistance in their countries **(B)**. It is important to note that West and East African countries are more prepared on AATr than their African neighbors in Central and Southern Africa, which all lie within the endemic regions of the continent.

Most experimental studies have been conducted in Europe, that is, France and the United Kingdom, while in Africa, only Nigeria and Kenya have strongly developed biomedical laboratories, which have attempted to conduct these studies (Figure 2A). Cross-sectional surveys are seldom conducted in African countries with no country reporting more than two studies over the last two decades (Figure 2B). This raises major challenges for trypanosomiasis control and eradication initiatives since the high prevalence of AAT on the African continent is mainly spread along the sub-Saharan belt, a region with continuous neglected clinical trials on AATr to date.

### 3.2. Community evidence on African animal trypanocide resistance

Here we report an AAT total random effect of 12.4% (95% CI: 7.6–18.2) using the randomized model (Table 3). The test for heterogenicity in these studies was *Q* (*df*); *95%CI, P-*value at 182.21(6), *I*^2^ = *96.71%, 95% CI: 94.97*–*97.85; P* < *0.0001*. To assess publication bias, Begg's test and Egger's test were used. Begg's test (correlations of proportions and meta-analysis weight) and Kendall's τ = 0.4286, *P* = 0.1765, while Egger's test (difference over standard error against one over standard error, that is, the regression analysis of study difference) was 3.6, 95% CI: −14.6 to 21.8, *P* = 0.6324, demonstrating a lack of publication bias in the studies assessed.

**Table 3:** Meta-analysis on the prevalence of African animal trypanocide resistance.

**Study**	**Sample size**	**Proportion (%)**	**95% CI**	**Weight (%)**	
				**Fixed**	**Random**
Afewerk et al.	484	17.15	13.90–20.81	9.91	14.27
Mungube et al.	796	15.70	13.24–18.42	16.29	14.52
Tchamja et al.	1,883	10.20	8.87–11.65	38.50	14.74
Mulandane et al.	467	22.91	19.18–27.00	9.56	14.25
Olila et al.	490	3.67	2.19–5.74	10.03	14.28
Mamoudou et al.	221	20.81	15.66–26.77	4.54	13.59
Kulohoma et al.	546	3.66	2.25–5.60	11.18	14.34
Total (fixed effects)	4,887	11.43	10.55–12.35	100	100
Total (random effects)	4,887	12.41	7.57–18.23	100	100

### 3.3. Preclinical studies on African animal trypanocide resistance

**Figure 3:**
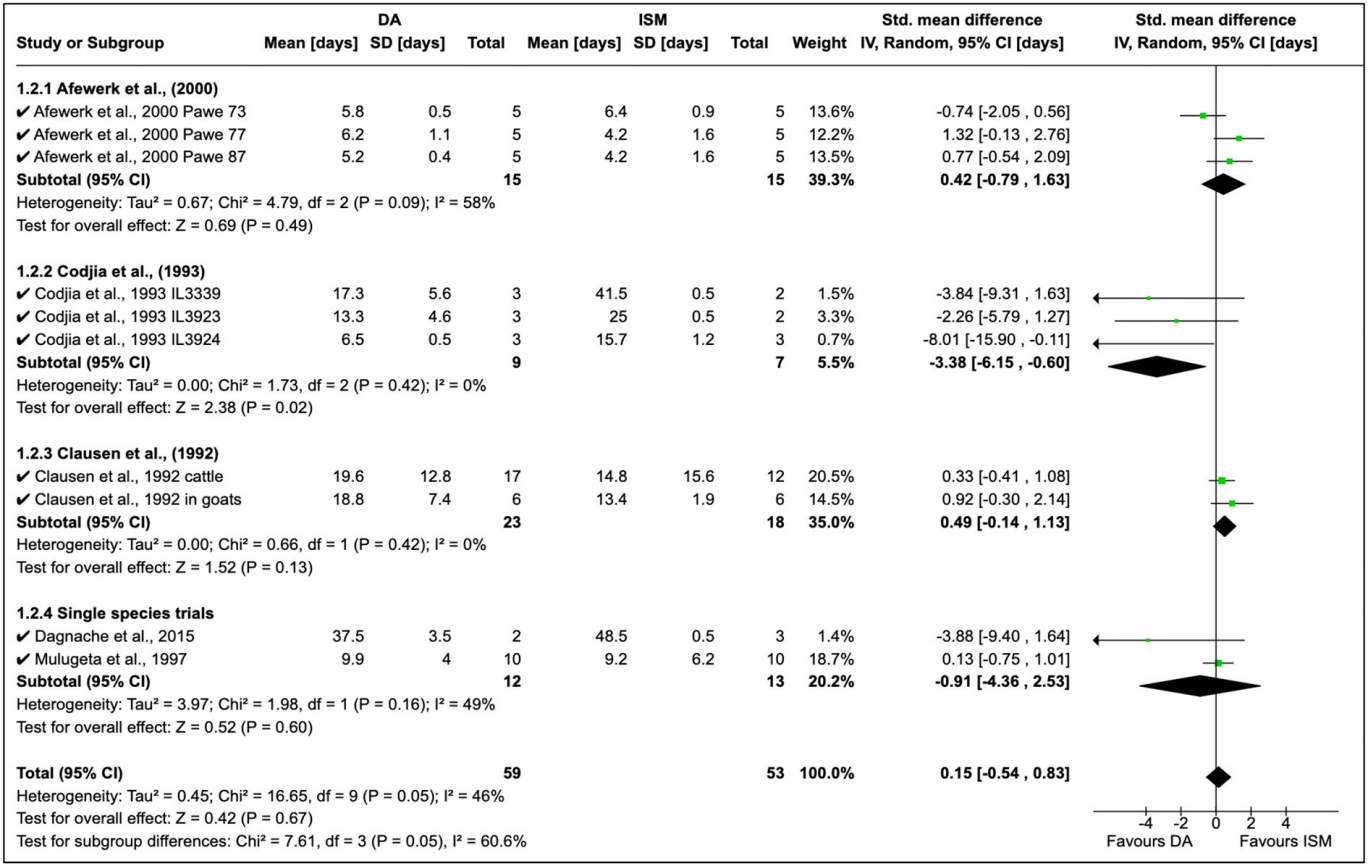
Mean relapse time in preclinical trials with diminazene aceturate and isometamidium chloride.

A total of seven quantitative studies were identified; however, only five studies were included in the analysis [eliminated (21) due to the long observation period and (41) who worked on only ISM]. The development of AATr has been investigated using DA at 3.5–7 mg/kg and ISM at 0.5–1.0 mg/kg (Figure 3). An experimental study by Afewerk et al. (22) conducted in Ethiopia showed relapse after 90 days with ISM. In this study (Figure 3, section 1.2.1), the sample size was small (*n* = 15), thus accounting for the moderate effect size in the study (*I*^2^ = 58%). Drug sensitivity showed that ISM relapse was higher than DA, although these findings were coincidental (RR = 0.42, 95% CI: −0.79, 1.63). In addition, the double resistant phenotype was genetically transferable to clones (A, B, and C), demonstrating genetic stability in the resistant genotype.

Cordjia et al. (23) demonstrated a high DA relapse although the reliability of this study was poor (*I*^2^ = 0%). In this study, three strains of *T. congolense* were used in goats; however, the high homogeneity (τ^2^ = 0%) showed these arose from the same population (Figure 3, Section 1.2.2). This was true since the field isolates were all from male and female East African cattle isolated in 1989 in Ethiopia. Clausen et al. (24) in Burkina Faso reported more ISM relapse than DA although these observations were also incidental due to the low variability in the study although authors worked on both cattle and goats (see Figure 3 where *T. congolense* and *T. vivax* isolates are used).

**Figure 4:**
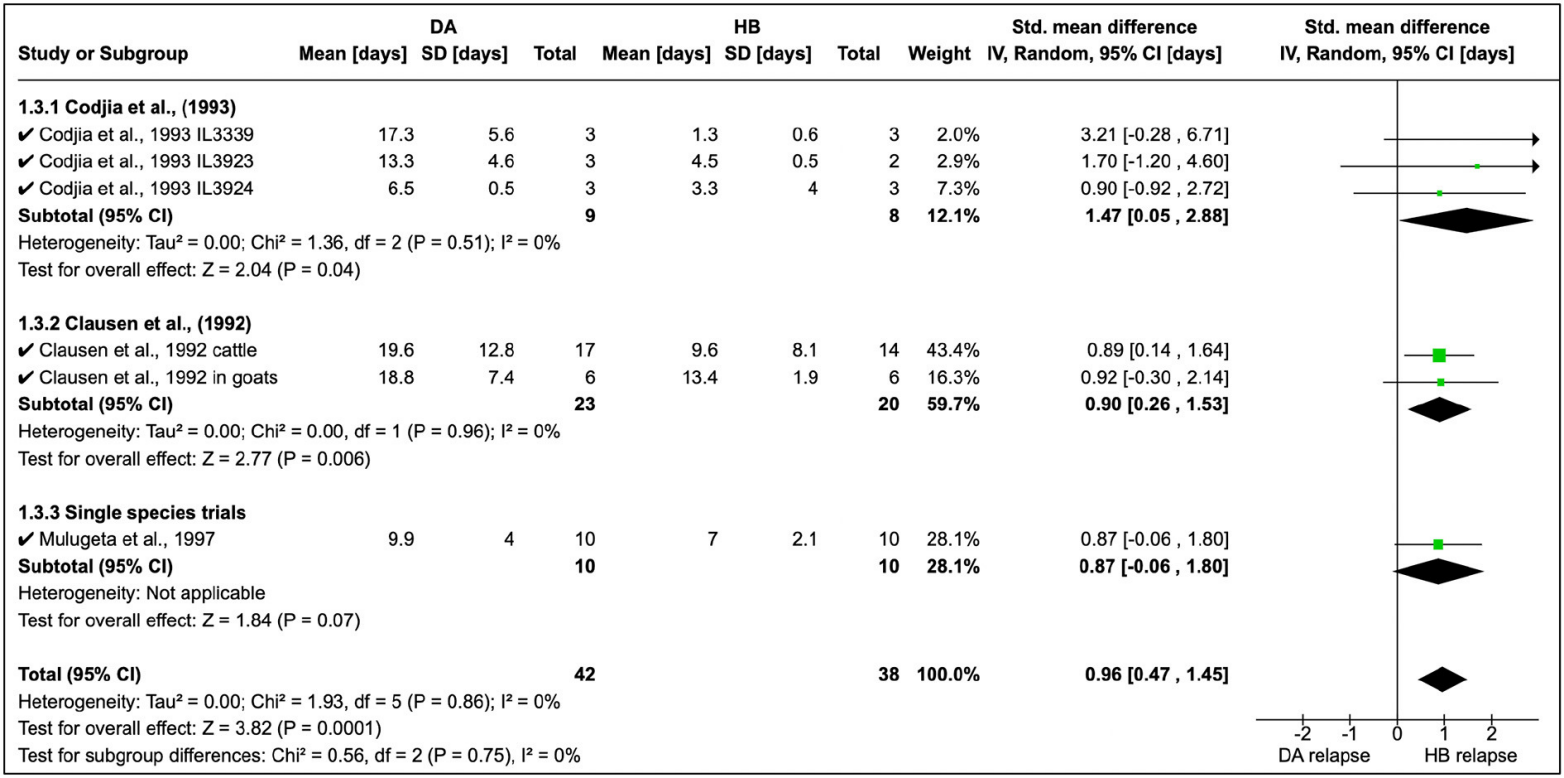
Mean relapse time in preclinical trials with diminazene aceturate and homidium bromide.

Cordjia et al. (23) also combined DA and HB and showed that relapse was highest with HB (SMD = 1.47, 95% CI: 0.05, 2.88), and similar findings (Figure 4) were reported by Clausen et al. (24) and Mulugeta et al. (26). The overall effect of these studies was identical (τ^2^ = 0.00) although these were conducted in different animal species (Figure 4).

**Figure 5:**
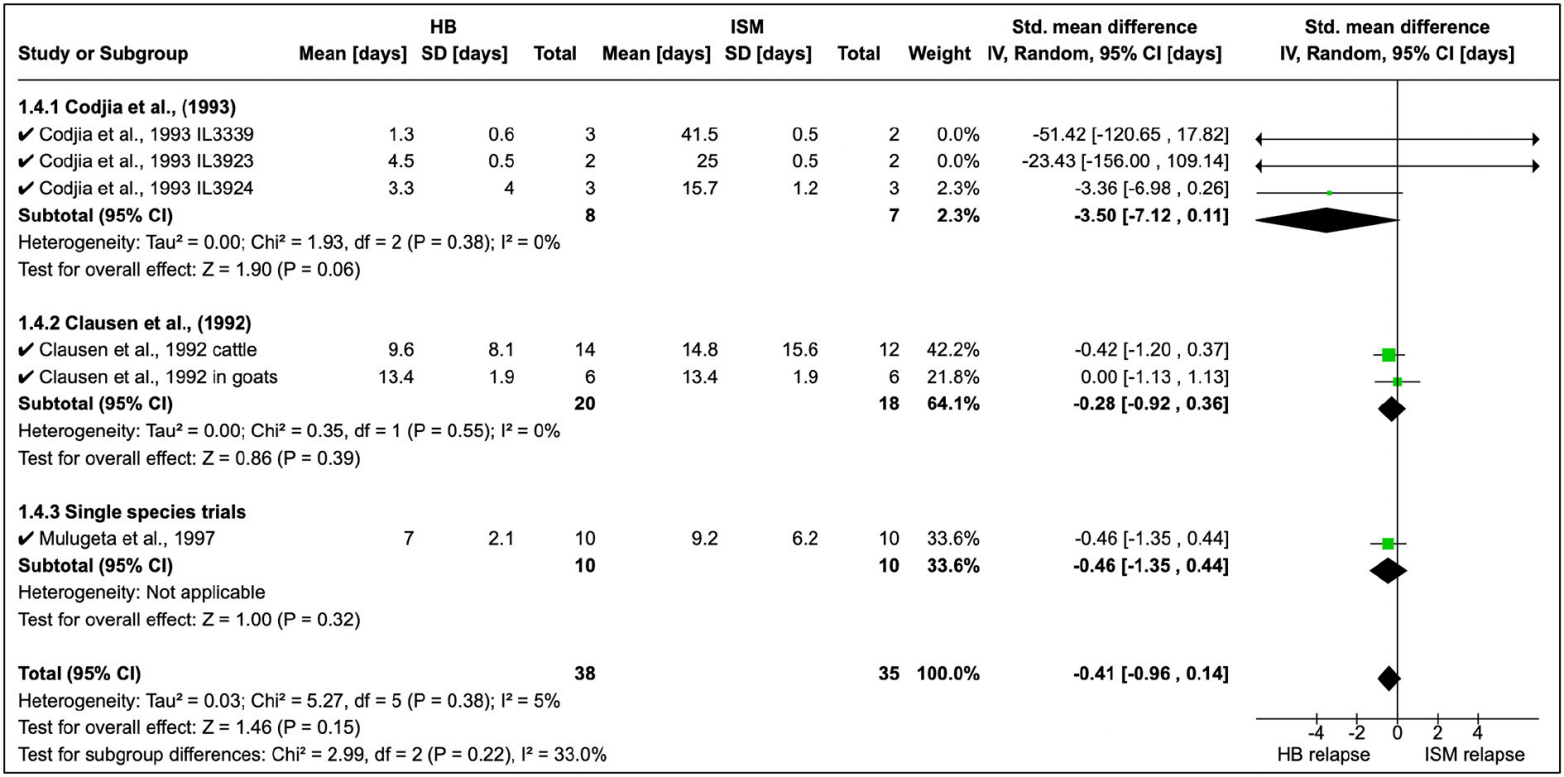
Mean relapse time in preclinical trials with homidium bromide and isometamidium chloride.

Homidium resistance in livestock was found to be higher than isometamidium (**Table 5**). Cordjia et al. (23) demonstrated that HB was a weaker drug than ISM against AATr phenotypes (see Section 1.4.1 in Figure 5) and this was in agreement with Clausen et al. (24). In addition, Mulugeta et al. (26) reproduced the same phenotypic characteristics showing that HB is a weaker (resistance higher) therapeutical option than ISM in Ethiopia using Boran (*Bos indicus*) calves infected with *T. congolense* strains. In addition, the same study showed that absorption of ISM was six times lower than the drug-sensitive phenotype, demonstrating the importance of drug-resistance mechanisms.

### 3.4. Clinical studies on African animal trypanocide resistance

**Figure 6:**
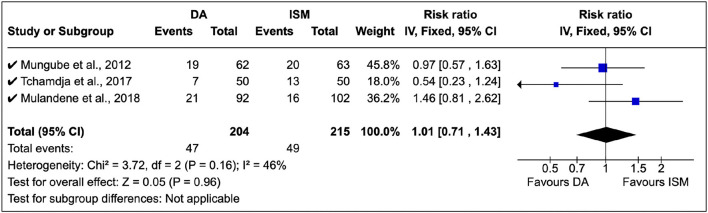
Clinical studies on African trypanocide resistance showing the number of animals that relapsed after 14 days.

A total of three surveys were identified in the study, and treatment relapse was the common approach used. Mungube et al. (5) showed that there was multiple resistance to DA and ISM in Mali (see Figure 6). Tchamdja (27) showed that DA resistance was most abundant in Togo, while Mulandane (28) reported a higher ISM relapse risk than DA in Mozambique. There was no common consensus on the shift between DA and ISM (RR = 1.01, 95% CI: 0.81, 2.62). In these clinical studies, there was a moderate level of homogeneity (*P* = 0.16).

In these studies, DA was administered at 3.5 mg/kg while ISM was at 0.5 mg/kg in line with the manufacturer's instructions.

### 3.5. Resistance markers screened in African animal trypanocide resistance

Mewamba et al. (29) found that the *Tb*AT1 gene is responsible for DA resistance, while de Koning and Jarvis (30) reported that P1/P2 was associated with DA resistance. In addition, methylglyoxal bis (guanylhydrazone) (MGBG) mitochondrial transport (32) was associated with diminazene resistance (Table 4). Resistance has also been associated with immune suppression of leukocytes in association with elevated serum liver enzymes (38).

**Table 4:** Resistance markers screened in African animal trypanocide resistance.

**Study**	**Country of the last author**	**Study was undertaken**	**Marker**	**Observations**
Mewamba et al. (29)	Cameroon	Cameroon	*Tb*AT1 followed by digestion of *Sfa* Nf1	40/45 had lost the gene
Koning and Jarvis (30)	United Kingdom	United Kingdom	P1/P2 transporters	P2-like transporters in the uptake of DA
Carter et al. (31)	United Kingdom	United Kingdom	P2	Diamidines are P2 transport substrates
Carruthers et al. (20)	United Kingdom	United Kingdom	*T. b. congolense* folate transporters I, II, and III	Mitochondrial membrane potential
Bacchi et al. (32)	Switzerland	Switzerland	Inhibition of polyamine biosynthesis	MGBG resembles diminazene. Even when MGBG used at 25 mg/kg for 3 days, was not curative for *T. b. brucei*
Zhang et al. (33)	France	France	Investigated diminazene, suramin, melCy, Qunapyramine, ISM	Minimal effective concentration (MEC) and maximum tolerable concentration (MTC)
			MEC and MTC vary with strains	
Gray and Peregrine (34)	Kenya	United Kingdom	MEC	Effective concentration *in vivo* may not work in cattle
Anene et al. (35)	United Kingdom	United Kingdom	MEC	Combinations of melarsen oxide cysteamine, diminazene aceturate or suramin with any of the candidate resistance modulators did not lead to any differences in the susceptibility pattern between sensitive and resistant *T. evansi* stocks studied
Sahin et al. (36)	France	France	Relapse	A standard dose of commercial ISM products contains < 0.1 mg/kg of the disubstituted compound which would be insufficient for a trypanocidal effect
Zhang et al. (37)	France	France	MEC	Field resistance can be reproduced in the lab and clones can remain sensitive
Akpa et al. (38)	Nigeria	Nigeria	ALT, AST, and WBC	Immune suppression by low WBC. Elevated serum liver enzymes

### 3.6. Rationale for increasing the dosage to combat trypanocide resistance

To address AATr, an increase in the treatment period has been explored; that is, Mungumbe (5) explored about maintaining the recommended dosage of 0.5 mg/kg and followed up the cattle for a month; however, this approach did not eliminate the development of resistance against DA. In addition, increasing the DA dosage by 3–4 times [see Ref. (24) on goats and Ref. (22) on rats] did not improve the therapeutical outcome in these patients (Table 5).

**Table 5:** Interventions to address trypanocide resistance in preclinical studies with dosages administered using different experimental models.

**Study**	**Parasite**	**Host**	**Drug and dosage**	**Intervention**	**Observation period (days)**	**Relapse/total**
Mungube et al. (5)	*T. congolense*	Cattle	ISM at 0.5 mg/kg	Increase treatment period	28	11/43
	*T. congolense*	Cattle	DA at 7 mg/kg	Overdose	14	7/20
Clausen et al. (24)	*T. congolense*	Goats	DA at 14 mg/kg in goats	Overdose	18	5/6
Clausen et al. (24)	*T. congolense*	Goats	DA at 17.5 mg/kg	Overdose	17	6/6
Clausen et al. (24)	*T. congolense*	Goats	Quinapyramine sulfate at 5.0 mg/kg	Overdose	13	8/8
Clausen et al. (24)	*T. congolense*	Goats	ISM at 1.0 mg/kg in goats	Overdose	13	5/6
Clausen et al. (24)	*T. congolense*	Goats	ISM at 2.0 mg/kg in goats	Overdose	21	2/6 (3 died)
Mulandane et al. (28)	*T. congolense*	Cattle	DA at 3.5 mg/kg and ISM at 0.5 mg/kg in cattle	Treatment swap	14	11/82 on DA and 9/102 on ISM
Afewerk et al. (22)	*T. congolense*	Rats	ISM at 1.0 mg/kg	Overdose and increase treatment period	90	7/30
Afewerk et al. (22)	*T. congolense*	Rats	DA at 28 mg/kg Pawe 73	Overdose	16	5/5
Afewerk et al. (22)	*T. congolense*	Rats	ISM at 4.0 mg/kg	Overdose	17	5/5
Afewerk et al. (22)	*T. congolense*	Rats	DA at 56 mg/kg	Overdose	NR	1/13
Afewerk et al. (22)	*T. congolense*	Rats	ISM at 16.0 mg/kg	Overdose	NR	1/15
ANENE et al. (39)	*T.b. brucei*	Dogs	Pentamidine at 4 mg/kg	Switch to pentamidine	10	0/6
Osman et al. (40)	*T. evansi*	Mice	Induced resistance at 0.5 mg/kg MelCy	Irradiation	1	Immune compromised induces resistance

KEY: NR = Not reported.

Several surveys explored the effect of increasing the dosage on treatment outcomes (Table 5). Treatment swaps (DA/ISM for ISM/DA) have also been attempted by Mulandane et al. (28) for an extended 2 weeks and these did not eliminate resistance against DA and ISM. In Nigeria, Anene et al. (39) assessed the quality of DA and found 2 out of 4 samples to contain < 95 and 90% of the stated constituency (Table 5), and cross-reactive resistance with pentamidines was reported in dogs. Furthermore, the continuous passage of *T. evansi* clones produced a resistant phenotype to melCyl (40), and this provided evidence that immunosuppressed hosts provide a conducive atmosphere for the development of drug resistance (Table 5).

## 4. Discussion

This study provides evidence on the low global interest in AATr clinical and preclinical trials following sporadic development of drug resistance in most livestock species, and this was in agreement with previous findings (42). In the African continent, there is a scarcity of information on AATr with only a couple of studies arising from West and East Africa, despite their interconnected livelihoods (especially livestock trade and free movement of animals), which act as conduits for infection and reinfection in neighboring countries. Laboratory studies have mainly been supported by the United Kingdom and France (98%) demonstrating their strategic importance for the strengthening of global health collaborations for the attainment of the WHO 2030 target since most of these surveys were conducted before the development of the sustainable developmental goals (SDGs). Developing countries, especially in Africa, continue to lag in biomedical research and routine monitoring of disease [little effort is taken on HAT (43) demonstrating a lack of commitment to AAT], demonstrating critical infrastructure challenges, justifying the need for collaborative research on the African continent (12). In particular, only one biomedical (preclinical) study was fully conducted in an African country (Nigeria) (38), demonstrating critical infrastructure weaknesses in the continent.

AATr has mainly been investigated using DA and ISM with a few studies working on HB. Here, we showed that trypanocide resistance profiles were in the order of HB > DA > ISM; however, the chronic lack of sufficient studies from the African continent implies that it is difficult to apply findings from this study across the entire African continent. Preclinical studies (22–24), which employed dosages at clinically acceptable dosages, demonstrated cross-reactive resistance to DA-ISM-HB although reasons for the development of these resistant phenotypes were not fully explored. This trend raises major threats to developing countries that have not received any capacity to develop alternative therapeutical options for the management of AATr. Furthermore, findings by Afewerk et al. (22) and Clausen et al. (24) showed that ISM relapse was a realistic threat, while Cordjia et al. (23) presented the only study that coincidentally reported DA relapse being higher than ISM. For curative purposes, DA is often preferred compared with ISM, which has both curative and prophylactic purposes and affordability, thus making it an easier drug to access and abuse. It is apparent that ISM is most preferred for the management of AATr, demonstrating its high contribution to drug resistance in this study.

Cross-resistance was also reported in these studies between DA and HB (23, 24, 26). Relapses due to HB were found to occur more often than those due to DA despite the fact these studies were conducted in varying animal species. Evidence from our study shows that the resistant phenotype may not be lost through passages in different animal species. Cross-species infections naturally occur in the wild [see Ref. (9) on wildlife species and Ref. (10) on small ruminants at the wildlife–human interface], so it can be implied that other livestock species do act as maintenance species responsible for the re-emergence of HAT. For example, most African countries have trypanosome control programs aimed at cattle while ignoring small ruminants, dogs, and other animals, which live in the same environment. These findings justify a revision in the current disease control strategies in several developing countries to eliminate and minimize maintenance hosts for reinfection.

**Figure 7:**
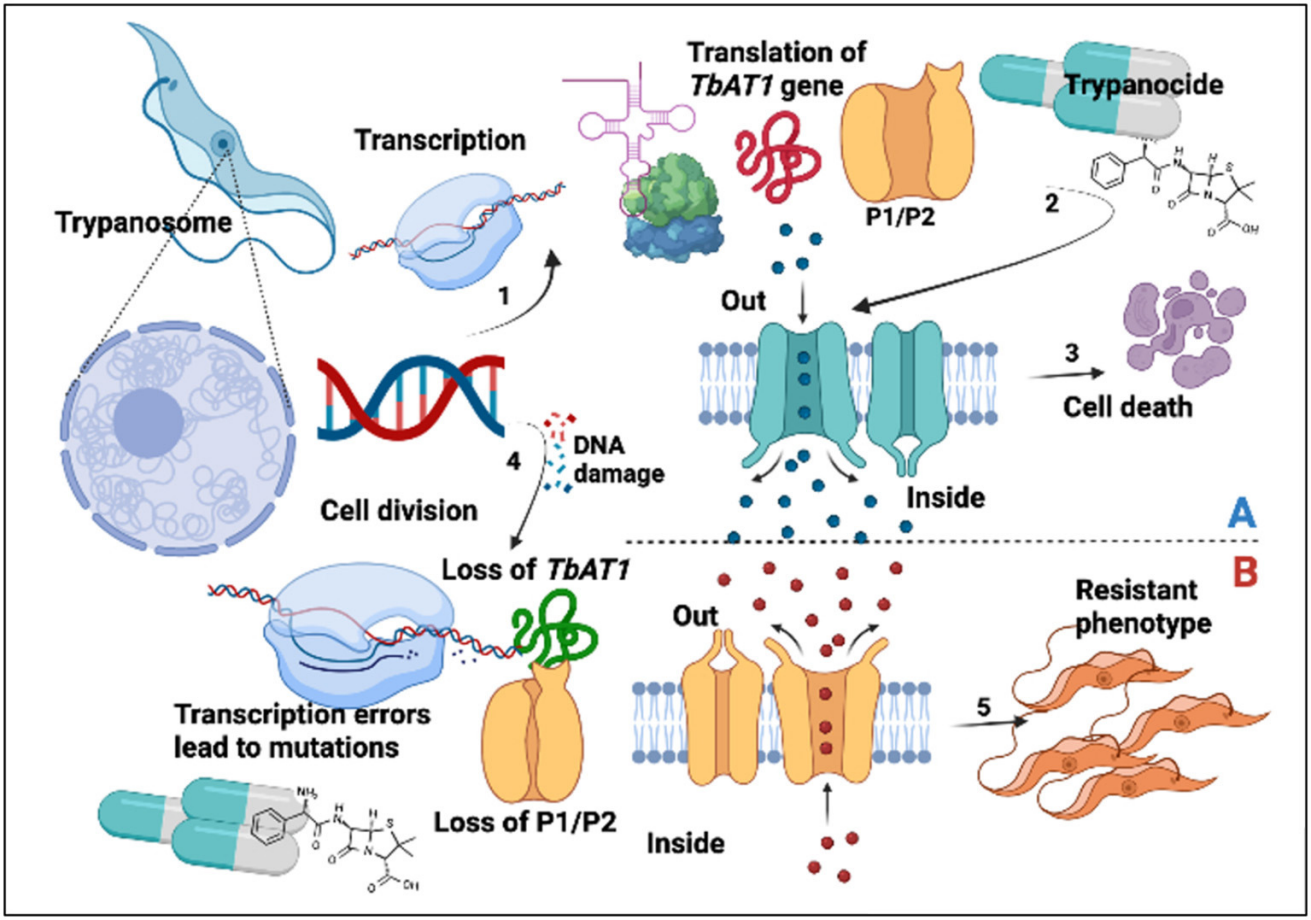
Trypanocide activity is favored by a healthy phenotype **(A)**. (1) Gene expression leading to production of *Tb*AT1/P2. Trypanocides (2) move into the cell to exert their effects leading to cell death (3). Resistant phenotype **(B)**. This arises following DNA damage (4), transcription errors, and subsequent loss of P1/P2 transporters. This subsequently disrupts trypanocide absorption into the parasite, leading to the establishment of a resistant phenotype (5).

The major AATr-responsible genes that have been investigated include *Tb*AT1, P1/P2 transporters, and folate transporters I, II, and III (20, 29–31), as well as host liver enzymes (38) and inhibitors of polyamine biosynthesis (32). Findings in this study are important since P2/*Tb*AT1 resistant genes continue to be essential for DA, ISM, and HB, which was in agreement with our previous study (17). Mutations in the aminopurine transporter *AT1* have been associated with reduced trypanocide absorption, while loss of *P2* favors the expression of more resistant phenotypes (6). Increased circulation of resistant genes in a community exerts selection pressure, which leads to the development of AATr (Figure 7).

The use of high-dosage regimens in several experimental studies [see Ref. (24) on goats and Ref. (22) on rats] failed to improve the clinical outcomes in cases of DA and ISM resistance. This provides a basis to discourage clinicians from doubling and increasing dosages once resistance has been reported against a trypanocide. Furthermore, interchanging medications and increasing the treatment period failed to provide any relief (5), providing a rationale for the promotion of evidence-based therapy while addressing drug resistance. AATr is also propagated by counterfeit trypanocides in circulation (39) and an immune-suppressed status of the host (40) demonstrating the importance of strong drug regulatory policies against fake medicines and farmer education to improve pharmacovigilance in affected communities.

## 5. Conclusion

African animal trypanocide resistance is a valid threat to the attainment of the WHO 2030 target. This situation has been created due to the limited number of therapeutical options available and limited epidemiological and biomedical studies to monitor drug efficacy in endemic communities. The development of AATr has heavily been associated with homidium bromide and isometamidium chloride, since they are routinely used for prophylaxis purposes; however, drug resistance of all available major trypanocides is cross-reactive. Interventions involving an increase in the dosage and treatment duration have not been able to improve the prognosis of affected animals. Further research to create localized genetic databases and libraries could pave the way toward further knowledge and research in developing countries for the effective monitoring of the AATr challenge and help to advise policy in resource-limited countries.

## Data availability statement

The datasets presented in this study can be found in online repositories. The names of the repository/repositories and accession number(s) can be found in the article/Supplementary material.

## Author contributions

KK and SW conceptualized the study. KK, SW, and EM conducted the study design. KK collected the data and conducted data analysis. All authors reviewed, approved the publication of the manuscript, and remain in agreement on all aspects of the work.
