# Supplementary material for: African animal trypanocide resistance: A systematic review and meta-analysis
Source: Front Vet Sci. 2023 Jan 4;9:950248. doi: 10.3389/fvets.2022.950248 (PMC9846564; doi:10.3389/fvets.2022.950248)
Supplement: Supplementary file 1 [file Table_1.DOCX]

**Supplementary file 1: Protocol for literature search with Databases**

The study explored 7 available databases to extract data on trypanocide resistance in AAT.

**2.1 CABI Abstracts**

CAB Abstracts <1946 to December Week 4 2021>

1 african trypanosomiasis/ or trypanosoma brucei/ or trypanosomiasis/ or tsetsefly-borne diseases/ 23469

2 bovine trypanosomiasis.mp. or Trypanosoma.od. or Trypanosoma brucei.od. 27016

3 tsetse fly.mp. [mp=abstract, title, original title, broad terms, heading words, identifiers, cabicodes] 5940

4 glossina.mp. [mp=abstract, title, original title, broad terms, heading words, identifiers, cabicodes] 5927

5 1 or 2 or 3 or 4 30413

6 trypanosoma brucei brucei.mp. 1286

7 exp trypanosoma vivax/ 1630

8 exp trypanosoma congolense/ 2374

9 exp trypanosoma evansi/ 2380

10 6 or 7 or 8 or 9 6547

11 5 and 10 5196

12 trypanocides/ or diminazene/ or homidium bromide/ or isometamidium chloride/ or melarsomine/ or quinapyramine/ 4129

13 trypano* resistance.mp. [mp=abstract, title, original title, broad terms, heading words, identifiers, cabicodes] 40

14 12 and 13 17

15 11 and 14 3

URL: https://www.ezproxy.is.ed.ac.uk/login?url=http://ovidsp.ovid.com/ovidweb.cgi?T=JS&NEWS=N&PAGE=main&SHAREDSEARCHID=65RCU8sv3asNGvYgeQU8LNFqpBJ1RSA3bcgonLCUArsSVclnJqvfG6LMSXUc27LdL

**2.2 MEDLINE**

Ovid MEDLINE(R) <1946 to December Week 4 2021>

1 african trypanosomiasis/ or trypanosoma brucei/ or trypanosomiasis/ or tsetsefly-borne diseases/ 15422

2 (bovine trypanosomiasis or Trypanosoma or Trypanosoma brucei).mp. [mp=title, abstract, original title, name of substance word, subject heading word, floating sub-heading word, keyword heading word, organism supplementary concept word, protocol supplementary concept word, rare disease supplementary concept word, unique identifier, synonyms] 31852

3 tsetse fly.mp. [mp=title, abstract, original title, name of substance word, subject heading word, floating sub-heading word, keyword heading word, organism supplementary concept word, protocol supplementary concept word, rare disease supplementary concept word, unique identifier, synonyms] 1104

4 glossina.mp. [mp=title, abstract, original title, name of substance word, subject heading word, floating sub-heading word, keyword heading word, organism supplementary concept word, protocol supplementary concept word, rare disease supplementary concept word, unique identifier, synonyms] 1857

5 1 or 2 or 3 or 4 35996

6 trypanosoma brucei brucei.mp. 7777

7 exp trypanosoma vivax/ 379

8 exp trypanosoma congolense/ 1011

9 trypanosoma evansi.mp. 807

10 6 or 7 or 8 or 9 9518

11 5 and 10 9518

12 trypanocides/ or diminazene/ or homidium bromide/ or isometamidium chloride/ or melarsomine/ or quinapyramine/ 9634

13 trypano* resistance.mp. [mp=title, abstract, original title, name of substance word, subject heading word, floating sub-heading word, keyword heading word, organism supplementary concept word, protocol supplementary concept word, rare disease supplementary concept word, unique identifier, synonyms] 27

14 12 and 13 11

15 11 and 14 4

URL: https://www.ezproxy.is.ed.ac.uk/login?url=http://ovidsp.ovid.com/ovidweb.cgi?T=JS&NEWS=N&PAGE=main&SHAREDSEARCHID=3xxz0F3SVHbdgsC2zCZDNA7XehdbMwovaQDeT0qzZsLu6wr0ohB1OHc9ALXsSqmJN

**2.3 EMBASE**

Embase <1974 to 2021 Week 51>

1 african trypanosomiasis/ or trypanosoma brucei/ or trypanosomiasis/ or tsetsefly-borne diseases/ 16493

2 (bovine trypanosomiasis or Trypanosoma or Trypanosoma brucei).mp. [mp=title, abstract, heading word, drug trade name, original title, device manufacturer, drug manufacturer, device trade name, keyword heading word, floating subheading word, candidate term word] 36779

3 tsetse fly.mp. [mp=title, abstract, heading word, drug trade name, original title, device manufacturer, drug manufacturer, device trade name, keyword heading word, floating subheading word, candidate term word] 2062

4 glossina.mp. [mp=title, abstract, heading word, drug trade name, original title, device manufacturer, drug manufacturer, device trade name, keyword heading word, floating subheading word, candidate term word] 1940

5 1 or 2 or 3 or 4 41001

6 trypanosoma brucei brucei.mp. 1580

7 exp trypanosoma vivax/ 746

8 exp trypanosoma congolense/ 1442

9 trypanosoma evansi.mp. 1057

10 6 or 7 or 8 or 9 4237

11 5 and 10 4237

12 trypanocides/ or diminazene/ or homidium bromide/ or isometamidium chloride/ or melarsomine/ or quinapyramine/ 7453

13 trypano* resistance.mp. [mp=title, abstract, heading word, drug trade name, original title, device manufacturer, drug manufacturer, device trade name, keyword heading word, floating subheading word, candidate term word] 33

14 12 and 13 8

15 11 and 14 3

URL: https://www.ezproxy.is.ed.ac.uk/login?url=http://ovidsp.ovid.com/ovidweb.cgi?T=JS&NEWS=N&PAGE=main&SHAREDSEARCHID=65RCU8sv3asNGvYgeQU8LMmwLn9N854HxtnaL0ywY3fZ5AfKtJTJbmCWFYpdC4Qnw

**2.4 CABI Global Health**

Global Health <1973 to 2021 Week 50>

1 african trypanosomiasis/ or trypanosoma brucei/ or trypanosomiasis/ or tsetsefly-borne diseases/ 18104

2 (bovine trypanosomiasis or Trypanosoma or Trypanosoma brucei).mp. [mp=abstract, title, original title, broad terms, heading words, identifiers, cabicodes] 32480

3 tsetse fly.mp. [mp=abstract, title, original title, broad terms, heading words, identifiers, cabicodes] 4504

4 glossina.mp. [mp=abstract, title, original title, broad terms, heading words, identifiers, cabicodes] 4481

5 1 or 2 or 3 or 4 34794

6 trypanosoma brucei brucei.mp. 1093

7 exp trypanosoma vivax/ 608

8 exp trypanosoma congolense/ 1017

9 trypanosoma evansi.mp. 613

10 6 or 7 or 8 or 9 2751

11 5 and 10 2751

12 trypanocides/ or diminazene/ or homidium bromide/ or isometamidium chloride/ or melarsomine/ or quinapyramine/ 2341

13 trypano* resistance.mp. [mp=abstract, title, original title, broad terms, heading words, identifiers, cabicodes] 21

14 12 and 13 9

15 11 and 14 2

URL: https://www.ezproxy.is.ed.ac.uk/login?url=http://ovidsp.ovid.com/ovidweb.cgi?T=JS&NEWS=N&PAGE=main&SHAREDSEARCHID=7LNdQ6zpBUBKJczRy7hMP8Gi9asbF6vyPx5HyUWcrOYki3r4ZZyjzPLdScC5p2l4N

**2.5 PubMed**

((African trypanosomiasis or trypanosoma brucei or trypanosomiasis/or tsetsefly-borne diseases OR bovine trypanosomiasis or Trypanosoma or Trypanosoma brucei OR tsetse fly OR glossina AND (("trypanosoma brucei brucei" OR "trypanosoma vivax" OR "trypanosoma congolense" OR "trypanosoma evansi") AND ("trypanocides or diminazene or "homidium bromide" or "isometamidium chloride/"or melarsomine or quinapyramine) AND trypanosome resistance") 2 papers

URL: https://pubmed.ncbi.nlm.nih.gov/?term=%28African+trypanosomiasis+or+trypanosoma+brucei+or+trypanosomiasis%2For+tsetsefly-borne+diseases+OR+bovine+trypanosomiasis+or+Trypanosoma+or+Trypanosoma+brucei+OR+tsetse+fly+OR+glossina+AND+%28%28%22trypanosoma+brucei+brucei%22+OR+%22trypanosoma+vivax%22+OR+%22trypanosoma+congolense%22+OR+%22trypanosoma+evansi%22%29+AND+%28%22trypanocides+or+diminazene+or+%22homidium+bromide%22+or+%22isometamidium+chloride%2F%22or+melarsomine+or+quinapyramine%29+AND+trypanosome+resistance%22%29&sort=relevance

**2.6 Web of Science**

[**((((ALL=(("african trypanosomiasis" or "trypanosoma brucei" or "trypanosomiasis" or "tsetsefly-borne diseases" OR "bovine trypanosomiasis "or "Trypanosoma" or "Trypanosoma brucei "OR "tsetse fly" OR glossina) AND trypanocides)) AND ALL=("african trypanosomiasis" or "trypanosoma brucei" or "trypanosomiasis" or "tsetsefly-borne diseases" OR "bovine trypanosomiasis " OR "Trypanosoma" OR "Trypanosoma brucei " OR "tsetse fly" OR glossina)) AND ALL=(("trypanosoma brucei brucei" or "trypanosoma vivax" or "trypanosoma congolense" or "trypanosoma evansi"))) AND ALL=((trypanocides or diminazene or hormidium bromide or "isometamidium chloride" or melarsamine or quinupramine))) AND ALL=(trypanocides resistance)** | 37 results](https://www.webofscience.com/wos/woscc/summary/5ea4e1c1-b718-40b6-b7d0-ed12448c2384-1c59d3e0/relevance/1)

URL: https://www.webofscience.com/wos/woscc/summary/5ea4e1c1-b718-40b6-b7d0-ed12448c2384-1c59d3e0/relevance/1

**2.7 Google Scholar**

Advanced search options were set to filter for the words “African trypanosomiasis” and the exact word or phrase “trypanocide resistance” was activated. Information was searched from OIE.INT and WHO.INT and FAO.INT as shown below;

OIE search

Results for **[african trypanosomiasis trypanosoma OR brucei OR brucei, OR trypanosoma OR vivax, OR trypanosoma OR congolense, OR trypanosoma OR evansi trypanocide resistance -human -african -trypanosomiasis site:oie.int](https://www.google.com/search?lr=&safe=images&hl=en-GB&as_qdr=all&sxsrf=AOaemvJmRsIMv1wP4l3lXMP00oy-3dVBLw:1641117731683&q=african+trypanosomiasis+trypanosoma+OR+brucei+OR+brucei,+OR+trypanosoma+OR+vivax,+OR+trypanosoma+OR+congolense,+OR+trypanosoma+OR+evansi+trypanocide+resistance+-human+-african+-trypanosomiasis+site:oie.int&sa=X&ved=2ahUKEwiuwrn755L1AhWOOcAKHTuHAzwQgwN6BAgBEAE)** (without quotes):

Result 2 papers

<https://rr-africa.oie.int/wp-content/uploads/2013/12/17-jamal_.pdf>

<https://www.oie.int/app/uploads/2021/03/2008-035-051-mariner-e.pdf>

WHO.INT

0

FAO.ORG

1 conference paper

<https://agris.fao.org/agris-search/search.do?recordID=FR2019119244>

AU.INT

0 papers

DNDI.ORG

2 papers

<https://dndi.org/wp-content/uploads/2009/07/ribeiro_chang.pdf>

<https://dndi.org/wp-content/uploads/2009/09/strub.pdf> (paper is on chagas)

KEMRI.ORG (Kenya)

0 papers

COCTU.ORG (Uganda)

0 papers

CDC

1 paper

<https://stacks.cdc.gov/view/cdc/68143/cdc_68143_DS1.pdf>

USAID

0 papers
